# Supplementary material for: HIVprotI: an integrated web based platform for prediction and design of HIV proteins inhibitors
Source: J Cheminform. 2018 Mar 9;10:12. doi: 10.1186/s13321-018-0266-y (PMC5845081; doi:10.1186/s13321-018-0266-y)
Supplement: Supplementary file 2 — Additional file 2. Source code of HIVProtI web server. [file 13321_2018_266_MOESM2_ESM.zip › HIVprotI_Source-code/hivprotI/weka-3-6-6/doc/weka/core/neighboursearch/TreePerformanceStats.html]

TreePerformanceStats


---


|  |  |  |
| --- | --- | --- |
| **Overview** \| **Package** \| **Class** \| **Tree** \| **Deprecated** \| **Index** \| **Help** \| **Weka's home** | |  |
| **PREV CLASS**   NEXT CLASS | **FRAMES**    **NO FRAMES**     **All Classes** |  |
| SUMMARY: NESTED \| FIELD \| CONSTR \| METHOD | DETAIL: FIELD \| CONSTR \| METHOD |  |


---


## weka.core.neighboursearch Class TreePerformanceStats

```
java.lang.Object
  weka.core.neighboursearch.PerformanceStats
      weka.core.neighboursearch.TreePerformanceStats
```

**All Implemented Interfaces:**: java.io.Serializable, AdditionalMeasureProducer, RevisionHandler

---

``` public class TreePerformanceStats extends PerformanceStats ```

The class that measures the performance of a tree based
nearest neighbour search algorithm.

**Version:**
:   $Revision: 1.2 $

**Author:**
:   Ashraf M. Kibriya (amk14[at-the-rate]cs[dot]waikato[dot]ac[dot]nz)

**See Also:**: Serialized Form

---

**Field Summary**

**Fields inherited from class weka.core.neighboursearch.PerformanceStats**

`m_CoordCount, m_MaxC, m_MaxP, m_MinC, m_MinP, m_PointCount, m_SumC, m_SumP, m_SumSqC, m_SumSqP`


**Constructor Summary**

`TreePerformanceStats()`
  
          Default constructor.


| **Method Summary** | |
| --- | --- |
| `java.util.Enumeration` | `enumerateMeasures()`             Returns an enumeration of the additional measure names. |
| `int` | `getMaxIntNodesVisited()`             returns the maximum of internal nodes visited. |
| `int` | `getMaxLeavesVisited()`             Returns the maximum number of leaves visited. |
| `double` | `getMeanIntNodesVisited()`             Returns the mean of internal nodes visited. |
| `double` | `getMeanLeavesVisited()`             Returns the mean of number of leaves visited. |
| `double` | `getMeasure(java.lang.String additionalMeasureName)`             Returns the value of the named measure. |
| `int` | `getMinIntNodesVisited()`             Returns the minimum of internal nodes visited. |
| `int` | `getMinLeavesVisited()`             Returns the minimum number of leaves visited. |
| `java.lang.String` | `getRevision()`             Returns the revision string. |
| `java.lang.String` | `getStats()`             Returns a string representation of the statistics. |
| `double` | `getStdDevIntNodesVisited()`             Returns the standard deviation of internal nodes visited. |
| `double` | `getStdDevLeavesVisited()`             Returns the standard deviation of leaves visited. |
| `int` | `getTotalIntNodesVisited()`             Returns the total number of internal nodes visited. |
| `int` | `getTotalLeavesVisited()`             Returns the total number of leaves visited. |
| `void` | `incrIntNodeCount()`             Increments the internal node count. |
| `void` | `incrLeafCount()`             Increments the leaf count. |
| `void` | `reset()`             Resets all internal fields/counters. |
| `void` | `searchFinish()`             Signals end of the nearest neighbour search. |
| `void` | `searchStart()`             Signals start of the nearest neighbour search. |

**Methods inherited from class weka.core.neighboursearch.PerformanceStats**

`getMaxCoordsPerPoint, getMaxPointsVisited, getMeanCoordsPerPoint, getMeanPointsVisited, getMinCoordsPerPoint, getMinPointsVisited, getNumQueries, getStdDevCoordsPerPoint, getStdDevPointsVisited, getTotalCoordsPerPoint, getTotalPointsVisited, incrCoordCount, incrPointCount, updatePointCount`

**Methods inherited from class java.lang.Object**

`equals, getClass, hashCode, notify, notifyAll, toString, wait, wait, wait`

**Constructor Detail**

### TreePerformanceStats

```
public TreePerformanceStats()
```

:   Default constructor.


**Method Detail**

### reset

```
public void reset()
```

:   Resets all internal fields/counters.

    :   **Overrides:**: `reset` in class `PerformanceStats`

---


### searchStart

```
public void searchStart()
```

:   Signals start of the nearest neighbour search.
    Initializes the stats object.

    :   **Overrides:**: `searchStart` in class `PerformanceStats`

---


### searchFinish

```
public void searchFinish()
```

:   Signals end of the nearest neighbour search.
    Calculates the statistics for the search.

    :   **Overrides:**: `searchFinish` in class `PerformanceStats`

---


### incrLeafCount

```
public void incrLeafCount()
```

:   Increments the leaf count.

---


### incrIntNodeCount

```
public void incrIntNodeCount()
```

:   Increments the internal node count.

---


### getTotalLeavesVisited

```
public int getTotalLeavesVisited()
```

:   Returns the total number of leaves visited.

    :   **Returns:**: The total number.

---


### getMeanLeavesVisited

```
public double getMeanLeavesVisited()
```

:   Returns the mean of number of leaves visited.

    :   **Returns:**: The mean number of leaves visited.

---


### getStdDevLeavesVisited

```
public double getStdDevLeavesVisited()
```

:   Returns the standard deviation of leaves visited.

    :   **Returns:**: The standard deviation of leaves visited.

---


### getMinLeavesVisited

```
public int getMinLeavesVisited()
```

:   Returns the minimum number of leaves visited.

    :   **Returns:**: The minimum number of leaves visited.

---


### getMaxLeavesVisited

```
public int getMaxLeavesVisited()
```

:   Returns the maximum number of leaves visited.

    :   **Returns:**: The maximum number of leaves visited.

---


### getTotalIntNodesVisited

```
public int getTotalIntNodesVisited()
```

:   Returns the total number of internal nodes visited.

    :   **Returns:**: The total number of internal nodes visited.

---


### getMeanIntNodesVisited

```
public double getMeanIntNodesVisited()
```

:   Returns the mean of internal nodes visited.

    :   **Returns:**: The mean number of internal nodes visited.

---


### getStdDevIntNodesVisited

```
public double getStdDevIntNodesVisited()
```

:   Returns the standard deviation of internal nodes visited.

    :   **Returns:**: The standard deviation of internal nodes visited.

---


### getMinIntNodesVisited

```
public int getMinIntNodesVisited()
```

:   Returns the minimum of internal nodes visited.

    :   **Returns:**: The minimum of internal nodes visited.

---


### getMaxIntNodesVisited

```
public int getMaxIntNodesVisited()
```

:   returns the maximum of internal nodes visited.

    :   **Returns:**: The maximum of internal nodes visited.

---


### enumerateMeasures

```
public java.util.Enumeration enumerateMeasures()
```

:   Returns an enumeration of the additional measure names.

    :   **Specified by:**: `enumerateMeasures` in interface `AdditionalMeasureProducer` **Overrides:**: `enumerateMeasures` in class `PerformanceStats`
    :   **Returns:**: An enumeration of the measure names.

---


### getMeasure

```
public double getMeasure(java.lang.String additionalMeasureName)
                  throws java.lang.IllegalArgumentException
```

:   Returns the value of the named measure.

    :   **Specified by:**: `getMeasure` in interface `AdditionalMeasureProducer` **Overrides:**: `getMeasure` in class `PerformanceStats`
    :   **Parameters:**: `additionalMeasureName` - The name of the measure to query for its value. **Returns:**: The value of the named measure. **Throws:**: `java.lang.IllegalArgumentException` - If the named measure is not supported.

---


### getStats

```
public java.lang.String getStats()
```

:   Returns a string representation of the statistics.

    :   **Overrides:**: `getStats` in class `PerformanceStats`
    :   **Returns:**: The statistics as string.

---


### getRevision

```
public java.lang.String getRevision()
```

:   Returns the revision string.

    :   **Specified by:**: `getRevision` in interface `RevisionHandler` **Overrides:**: `getRevision` in class `PerformanceStats`
    :   **Returns:**: the revision


---


|  |  |  |
| --- | --- | --- |
| **Overview** \| **Package** \| **Class** \| **Tree** \| **Deprecated** \| **Index** \| **Help** \| **Weka's home** | |  |
| **PREV CLASS**   NEXT CLASS | **FRAMES**    **NO FRAMES**     **All Classes** |  |
| SUMMARY: NESTED \| FIELD \| CONSTR \| METHOD | DETAIL: FIELD \| CONSTR \| METHOD |  |


---
